# Re-evaluating the role of natural killer cells in innate resistance to herpes simplex virus type 1

**DOI:** 10.1186/1743-422X-2-56

**Published:** 2005-07-17

**Authors:** William P Halford, Jennifer L Maender, Bryan M Gebhardt

**Affiliations:** 1 Dept of Veterinary Molecular Biology, Montana State University, Bozeman, MT, USA; 2 Dept of Dermatology, Baylor College of Medicine, Houston, TX, USA; 3 Dept of Ophthalmology, Louisiana State University Health Sciences Center, New Orleans, LA USA

## Abstract

**Background:**

Interferon-γ acts to multiply the potency with which innate interferons (α/β) suppress herpes simplex virus type 1 (HSV-1) replication. Recent evidence suggests that this interaction is functionally relevant in host defense against HSV-1. However, it is not clear which WBCs of the innate immune system, if any, limit HSV-1 spread in an IFN-γ dependent manner. The current study was initiated to determine if natural killer (NK) cells provide innate resistance to HSV-1 infection, and if so to determine if this resistance is IFN-γ-dependent.

**Results:**

Lymphocyte-deficient *scid *or *rag2*^-/- ^mice were used to test four predictions of the central hypothesis, and thus determine if innate resistance to HSV-1 is dependent on **1. **NK cell cytotoxicity, **2. **NK cells, **3. **WBCs, or **4. **the IFN-activated transcription factor, Stat 1. Loss of NK cell cytotoxic function or depletion of NK cells had no effect on the progression of HSV-1 infection in *scid *mice. In contrast, viral spread and pathogenesis developed much more rapidly in *scid *mice depleted of WBCs. Likewise, loss of Stat 1 function profoundly impaired the innate resistance of *rag2*^-/- ^mice to HSV-1.

**Conclusion:**

Lymphocyte-deficient mice possess a very tangible innate resistance to HSV-1 infection, but this resistance is not dependent upon NK cells.

## Background

Severe infections with herpesviruses such as herpes simplex virus type 1 (HSV-1) have been observed in natural killer (NK) cell-deficient individuals [1-3]. This observation has fostered the belief that NK cells play a central role in innate resistance to HSV-1 infection. This hypothesis is further supported by the mechanism of action of the viral ICP47 protein. ICP47 binds the cellular antigen transporter, TAP1, and thus prevents MHC class I molecules from being transported to the surface of HSV-1 infected cells [4]. This inhibition of MHC class I transport appears to explain the long recognized fact that HSV-1 infection renders cultured cells vulnerable to NK cell-mediated lysis [5-7]. Indeed, expression of ICP47 is sufficient, in and of itself, to downregulate MHC class I and induce NK cell-mediated lysis of human cells [8]. Numerous *in vitro *and *in vivo *studies also support the tenet that NK cells play an integral role in innate resistance to HSV-1 infection [9-13].

Against this background, it is not surprising that most current texts and reviews indicate that NK cells are essential for host resistance to HSV-1 infection [14-18]. However, this tenet is based upon equivocal evidence. A handful of animal studies from the last 25 years indicate that NK cells are not essential for host resistance to HSV-1 [19-21]. More recently, a similar conclusion was reached based on the comparison of HSV-1 infection in *rag2*^-/- ^mice versus *rag2*^-/- ^*γ_c_*^-/- ^mice [22]. However, loss of γ_c _not only prevents NK cell development, but also renders mice null for the function of interleukins (IL)-2,-4,-7,-9,-15, and -21. Given its pleiotropic effects [23-25], the γ_c_^-/- ^mutation does not provide a compelling basis for drawing inferences about any one component of the innate immune system. Numerous NK cell studies are confounded by similar caveats. For example, NK cell depletion has been found to impair host resistance to HSV-1 infection [12,26], but activated T cells also express ''NK cell'' markers [27]. Therefore, the effect of anti-asialo GM1 and anti-NK1.1 antibodies on host resistance to HSV-1 may be due, at least in part, to their capacity to blunt the T cell response to viral infections [27].

Interferon (IFN)-γ multiplies the potency with which the innate IFNs, IFN-α and/or IFN-β, suppress HSV-1 replication [28]. This cooperative inhibition by IFN-α/β and IFN-γ effectively prevents virus-infected cells from synthesizing new HSV-1 virions [29]. The profoundly accelerated rate of HSV-1 spread in receptor-deficient mice suggests that the interaction between the IFN-α/β-and IFN-γ-signaling pathways is functionally relevant in innate resistance to HSV-1 [22,30]. Consistent with this hypothesis, IFN-γ expression is evident in HSV-1 infected tissues just 24 hours post inoculation (p.i.; Fig. 7 of Ref. [31]). T cells, NK cells, and professional antigen-presenting cells (APCs) are the primary IFN-γ-producers in the body [32,33]. CD8^+ ^T cells play a major role in immune surveillance of HSV-1 latently infected ganglia, and can directly suppress HSV-1 reactivation in neurons in a manner that is MHC class I-restricted and IFN-γ-dependent [34-38]. However, it is unknown if NK cells and/or professional APCs confer innate resistance to HSV-1 infection via the secretion of IFN-γ at early times p.i.

**Figure 7:**
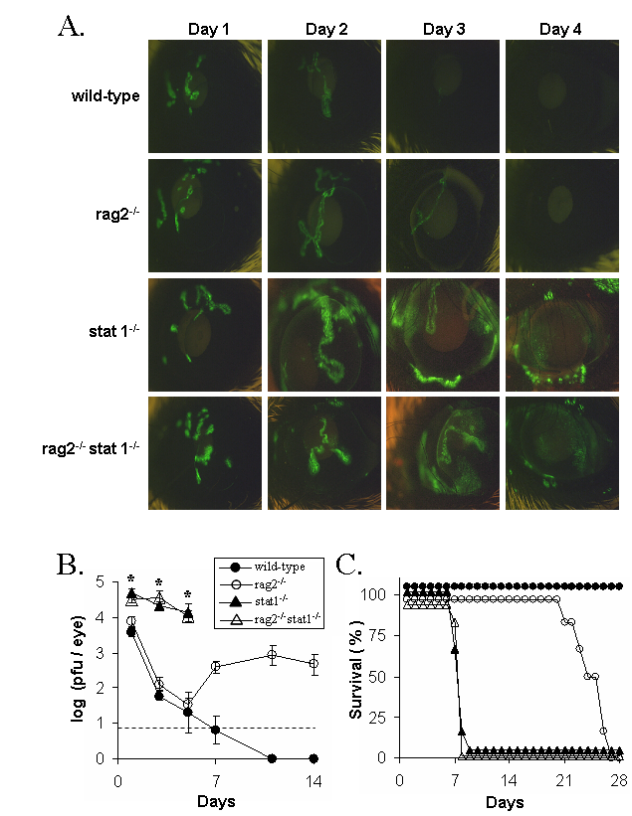
**Effect of Stat 1 on innate resistance to HSV-1**. Wild-type (strain 129) mice, *rag2*^-/- ^mice, *stat1*^-/- ^mice, and *rag2*^-/-^*stat1*^-/- ^mice were inoculated with 2 × 10^5 ^pfu/eye of HSV-1 strain KOS-GFP. **A. **Eyes of KOS-GFP-infected mice on days 1, 2, 3, and 4 p.i (4× magnification, illuminated with 360–400 nm light which excites GFP). A representative mouse from each group was sequentially imaged on days 1 through 4 p.i. **B. **Replication of HSV-1 strain KOS-GFP in the eyes of mice (mean ± SEM; n= 6; dashed line denotes lower limit of detection). Asterisks denote times at which *stat1*^-/- ^mice shed more virus than *stat1*^+/+ ^mice (p < 0.05, ANOVA and Tukey's post hoc t-test). **C. **Duration of survival of HSV-1 infected mice (n = 6 per group).

The following study was initiated to determine if NK cells provide innate resistance to HSV-1 infection via their capacity to rapidly deliver IFN-γ to sites of viral replication. *Scid *or *rag2*^-/- ^mice were used to test four predictions that follow from this central hypothesis. Specifically, experiments were performed to determine if innate resistance to HSV-1 is dependent on **1. **NK cell cytotoxicity, **2. **NK cells, **3. **WBCs, or **4. **the IFN-activated transcription factor, Stat 1 [39,40]. The use of lymphocyte-deficient mice assured that this analysis of innate resistance was not confounded by the dominant effects of the adaptive immune response. The results demonstrate that although *scid *and *rag2*^-/- ^mice possess a measurable resistance to HSV-1, this innate resistance is not dependent upon NK cells.

## Results

### Immune status of BALB/c scid mice

**Figure 1:**
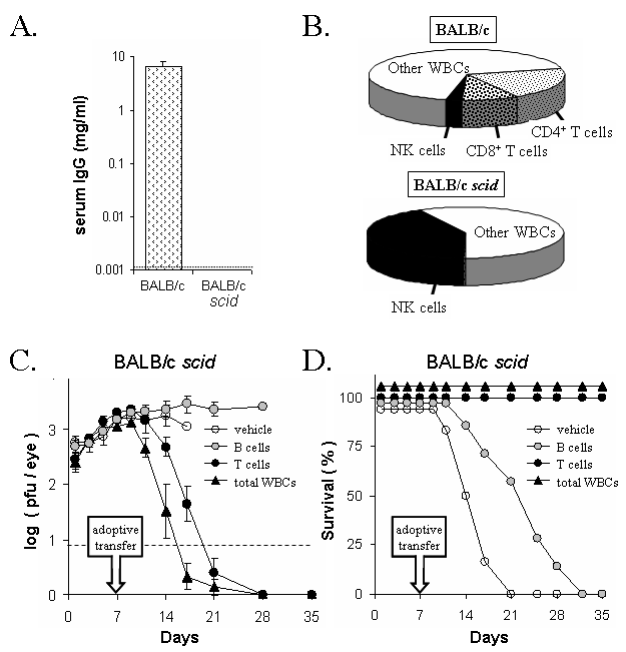
**Immune status of BALB/c *scid *mice**. **A. **ELISA measurement of serum IgG levels in BALB/c and BALB/c *scid *mice (n = 5 per group; dashed line denotes lower limit of detection). **B. **Flow cytometric measurement of the abundance of CD4^+ ^T cells, CD8^+ ^T cells, and CD3^- ^CD49b^+ ^NK cells in the spleens of BALB/c versus *scid *mice (n = 10 per group). "Other WBCs" refers to the fraction of spleen cells not labeled by antibodies against CD3, CD4, CD8, and CD49b. **C and D. **Effect of adoptively transferred naïve lymphocytes on *scid *mouse resistance to HSV-1. **C. **Viral titers per eye (dashed line denotes lower limit of detection) and **D. **duration of survival of *scid *mice inoculated with 2 × 10^5 ^pfu/eye HSV-1 strain KOS. On day 7 p.i., *scid *mice (n = 8 per group) received an i.v. injection of medium (**vehicle**) or medium containing 5 × 10^6 ^**B cells**, **T cells**, or unfractionated WBCs (**total WBCs**) obtained from naïve BALB/c donors.

Lymphocyte maturation is not completely blocked in some strains of *scid *mice [41-43]. Thus, B and T lymphocyte function were evaluated in *scid *mice. Assessment of B cell function indicated that BALB/c mice had serum IgG levels of 6.4 ± 1.3 mg/ml, whereas serum IgG was undetectable in *scid *mice (Fig. 1A). Flow cytometric analysis indicated that BALB/c mice contained an average 110 million WBCs per spleen, of which 21% were CD4^+ ^T cells, 10% were CD8^+ ^T cells, and 2.5% were CD3^- ^CD49b^+ ^NK cells (Fig. 1B). In contrast, *scid *mice contained an average 8 million WBCs per spleen, of which <0.1% were CD4^+ ^or CD8^+ ^T cells and 45% were CD3^- ^CD49b^+ ^NK cells (Fig. 1B).

Adoptive transfer was performed to verify that adaptive resistance to HSV-1 could be restored to *scid *mice. Following ocular inoculation with HSV-1 strain KOS, *scid *mice shed high titers of virus between 1 and 7 days p.i. (Fig. 1C). On day 7 p.i., *scid *mice were i.v. administered either **a. **vehicle, **b. **total WBCs, **c. **purified B cells, or **d. **purified T cells from naïve BALB/c donors (Fig. 1C). Vehicle-treated *scid *mice continued to shed high levels of virus (Fig. 1C) and succumbed to the infection within 17 ± 2 days p.i. (Fig. 1D). *Scid *mice reconstituted with total WBCs shed 30-fold less virus than vehicle-treated controls on day 14 p.i. (Fig. 1C) and 8 of 8 survived the infection (Fig. 1D). *Scid *mice reconstituted with purified B cells eventually died, but the mean time of survival was increased to 22 ± 3 days (Fig. 1D). Reconstitution with purified T cells controlled HSV-1 infection in 8 of 8 *scid *mice, but viral shedding continued for ~3 days longer than *scid *mice reconstituted with total WBCs (Fig. 1C). Thus, all measures indicated that *scid *mice are effectively devoid of B and T lymphocyte function.

### Innate resistance to HSV-1 is not dependent on NK cell cytotoxicity

**Figure 2:**
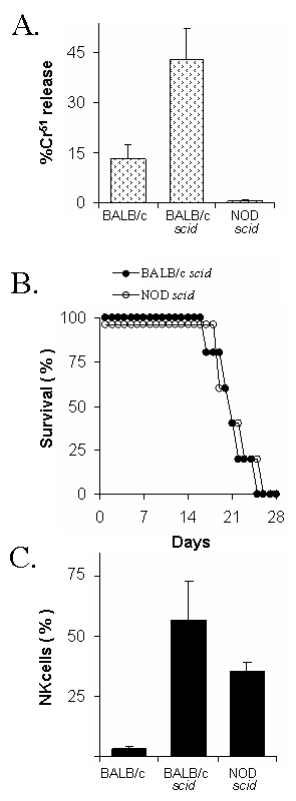
**Innate resistance to HSV-1 is not dependent on NK cell cytotoxicity**. **A. **Cytotoxic activity of WBCs from BALB/c, BALB/c *scid*, or NOD *scid *mice, as determined by percent maximum ^51^Cr release achieved when 10^4 ^YAC-1 (target) cells were incubated with 250,000 spleen WBCs (n = 3 per group). **B. **Duration of survival of BALB/c *scid *mice and NOD *scid *mice following ocular inoculation with 2 × 10^5 ^pfu/eye HSV-1 strain KOS (n = 5 per group). **C. **NK cell frequency in the spleens of BALB/c, BALB/c *scid*, or NOD *scid *mice (n = 2 per group).

To determine if innate resistance to HSV-1 is dependent on NK cell cytotoxic function, infection with HSV-1 strain KOS was compared in BALB/c *scid *mice versus non-obese diabetic (NOD) *scid *mice. Consistent with previous reports [44,45], WBCs isolated from the spleens of NOD *scid *mice were functionally deficient in NK cell cytotoxic activity relative to BALB/c mice and BALB/c *scid *mice (Fig. 2A). Following ocular inoculation with 2 × 10^5 ^pfu/eye, HSV-1 strain KOS replicated to high and equivalent titers in BALB/c *scid *mice and NOD *scid *mice between 1 and 14 days p.i. (not shown). No differences were observed in the progression of viral pathogenesis or the duration of survival of BALB/c *scid *mice versus NOD *scid *mice (Fig. 2B). Flow cytometry demonstrated that approximately one-third of the peripheral WBCs of NOD *scid *mice possessed the CD3^- ^CD49b^+ ^phenotype of NK cells (Fig. 2C) [46,47]. Thus, despite the lack of *in vitro *cytotoxic activity (Fig. 2A), NOD *scid *mice still possessed significant numbers of NK cells that could control HSV-1 infection via other mechanisms (e.g., IFN-γ secretion).

### Innate resistance to HSV-1 is not dependent on NK cells

**Figure 3:**
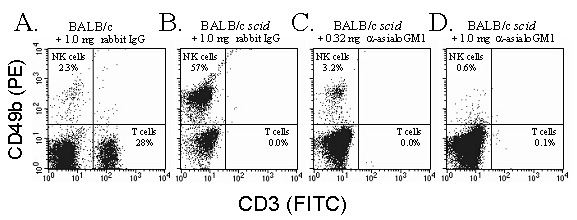
**Efficacy of NK cell depletion with anti-asialo GM1 antibody**. The frequency of CD3^- ^CD49b^+ ^NK cells in the spleens of **A. **BALB/c mice and **B. **BALB/c *scid *mice that received i.p. injections of 1.0 mg per day control rabbit IgG, as compared to *scid *mice treated with **C. **0.32 or **D. **1.0 mg per day of rabbit anti-asialo GM1. Mice were treated with antibody on Days 0 and 3, and spleen WBCs were isolated on Day 4 for flow cytometric analysis with FITC-labeled anti-CD3 and PE-labeled anti-CD49b. The frequency of NK cells (upper left quadrant) and CD3^+ ^T cells are indicated on each graph. Results are representative of three independent experiments.

Preliminary experiments indicated that two treatments with 0.32 or 1.0 mg rabbit anti-asialo GM1 reduced the number of NK cells in BALB/c *scid *mouse spleens by >10- and >50-fold, respectively, whereas control rabbit IgG produced no such effect (Fig. 3). Thus, anti-asialo GM1 antibody was used to determine if NK cells are necessary for innate resistance to HSV-1.

**Figure 4:**
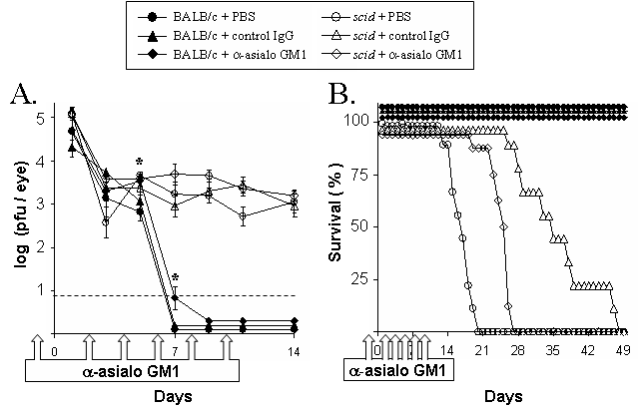
**Effect of NK cell depletion on innate resistance to HSV-1**. BALB/c mice and BALB/c *scid *mice, inoculated with 2 × 10^5 ^pfu/eye HSV-1 strain KOS, received i.p. injections of PBS, control IgG or anti-asialo GM1 (1.2 mg) on days -1, 2, 4, 6, 8 and 10 p.i. **A. **Viral replication in the eyes of BALB/c mice and *scid *mice treated with PBS, control IgG or anti-asialo GM1 (mean ± SEM; n = 9; dashed line denotes lower limit of detection). Asterisks denote times at which anti-asialo GM1-treated BALB/c mice shed more virus than PBS-treated BALB/c mice (p < 0.05, ANOVA and Tukey's post hoc t-test). **B. **Duration of survival of HSV-1 infected BALB/c mice and *scid *mice treated with PBS, control IgG or anti-asialo GM (n = 9 per group).

BALB/c mice and BALB/c *scid *mice were treated with PBS, control IgG, or anti-asialo GM1 and were inoculated with 2 × 10^5 ^pfu/eye of HSV-1 strain KOS. In BALB/c mice, KOS replicated to similar viral titers in mice treated with PBS, control rabbit IgG, or anti-asialo GM1, with one notable exception (Fig. 4A). On days 5 and 7 p.i., BALB/c mice treated with rabbit anti-asialo GM1 shed ~5-fold more virus than PBS-treated controls (Fig. 4A; p < 0.05, denoted by asterisks). Between days 9 and 14 p.i., viral shedding was detected in none of the BALB/c mice (Fig. 4A). Likewise, viral pathogenesis was limited, and 100% of BALB/c mice survived infection with HSV-1 strain KOS (Fig. 4B).

BALB/c *scid *mice shed infectious KOS continuously during the 14-day sampling period (Fig. 4A). Treatment with rabbit anti-asialo GM1 had no effect on the titers of infectious KOS recovered from the eyes of BALB/c *scid *mice between 1 and 14 days p.i. (Fig. 4A). *Scid *mice treated with PBS survived for 16 ± 1 days p.i. (Fig. 4B). Treatment with rabbit anti-asialo GM1 did not shorten the duration of survival of KOS-infected *scid *mice (Fig. 4B). Paradoxically, treatment with rabbit anti-asialo GM1 or control rabbit IgG increased the duration of survival of KOS-infected BALB/c *scid *mice to 24 ± 1 and 35 ± 3 days p.i., respectively (Fig. 4B; p <0.001). Multiple experiments confirmed this unexpected effect that rabbit immunoglobulin (with no reactivity against HSV-1) prolonged the survival of KOS-infected *scid *mice. The interpretation of these data was complicated by this caveat. However, it was clear that NK cell depletion did not fundamentally alter the progression of HSV-1 infection in *scid *mice during the first week p.i.

### Innate resistance to KOS is dependent on peripheral WBCs

**Figure 5:**
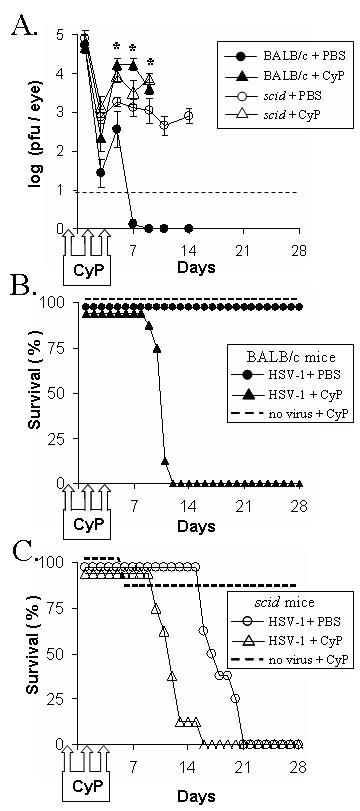
**Effect of WBC depletion on innate resistance to HSV-1**. BALB/c mice and BALB/c *scid *mice, inoculated with 2 × 10^5 ^pfu/eye HSV-1 strain KOS, received i.p. injections of PBS or cyclophosphamide (CyP; 125 mg/kg) on days -1, 1, and 3 p.i. Uninfected BALB/c mice and uninfected *scid *mice received i.p. injections of CyP at the same time points (n = 8 per group). **A. **Viral replication in the eyes of BALB/c mice and *scid *mice treated with PBS or CyP (mean ± SEM; n = 8; dashed line denotes lower limit of detection). Asterisks denote times at which CyP-treated BALB/c mice shed more virus than PBS-treated BALB/c mice (p < 0.05, ANOVA and Tukey's post hoc t-test). **B and C. **Duration of survival of HSV-1 infected **B. **BALB/c mice and **C. ***scid *mice treated with PBS or CyP versus uninfected, CyP-treated controls (n = 8 per group).

Cyclophosphamide (CyP) is an alkylating agent that is rapidly converted *in vivo *into metabolites that cause lethal DNA damage in dividing cells [48,49], and transiently reduce peripheral WBC counts by ~90% in mice [31]. To determine if WBCs are necessary for innate resistance to HSV-1, BALB/c mice and *scid *mice were treated with PBS or CyP and were inoculated with 2 × 10^5 ^pfu/eye of HSV-1 strain KOS. On day 4 p.i., peripheral WBC counts (WBCs per ml × 10^6^) in each group were, as follows: BALB/c + PBS = 6.6 ± 0.5; *scid *+ PBS = 2.3 ± 0.2; BALB/c + CyP = 0.8 ± 0.1; and *scid *+ CyP = 0.4 ± 0.1. Similar viral titers were recovered from the eyes of all mice at 24 hours p.i. (Fig. 5A). However, BALB/c mice treated with CyP shed 30- to 1000-fold more virus than PBS-treated BALB/c mice between 5 and 9 days p.i. (Fig. 5A; p < 0.05, denoted by asterisks). Likewise, CyP-treated *scid *mice shed 2- to 7-fold more virus than PBS-treated *scid *mice between 5 and 9 days p.i. (Fig. 5A). Viral titers were not determined in CyP-treated mice on 11 and 14 days p.i. because the extent of ocular pathogenesis precluded a reliable measurement.

BALB/c mice treated with PBS uniformly survived ocular HSV-1 infection (Fig. 5B). In contrast, 0% of CyP-treated BALB/c mice survived HSV-1 infection (Fig. 5B). The death of these mice was not a direct consequence of CyP's toxicity, because 100% of uninfected BALB/c controls survived the same course of CyP treatment (Fig. 5B). PBS-treated *scid *mice survived ocular inoculation with HSV-1 strain KOS for 18 ± 1 days (Fig. 5C). In contrast, CyP-treated *scid *mice succumbed to HSV-1 infection within 12 ± 1 days (Fig. 5C; p < 0.001). This reduced duration of survival was not a direct consequence of CyP's toxicity, because 7 of 8 uninfected *scid *mice survived CyP treatment (Fig. 5C). Therefore, depletion of total WBCs in *scid *mice was correlated with decreased innate resistance to HSV-1 infection.

### Effect of NK cell versus WBC depletion on innate resistance to HSV-1

**Figure 6:**
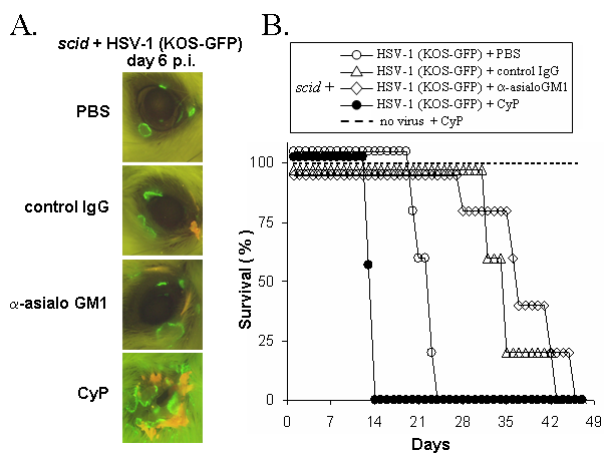
**Effect of NK cell versus WBC depletion on innate resistance to HSV-1**. BALB/c *scid *mice, inoculated with 2 × 10^5 ^pfu/eye HSV-1 strain KOS-GFP, received i.p. injections of PBS, control IgG or anti-asialo GM1 (1.7 mg) on days -1, 2, 5 and 9 p.i. Cyclophosphamide (CyP; 125 mg/kg) was administered on days -1, 1, and 3 p.i. **A. **Eyes of KOS-GFP-infected *scid *mice on day 6 p.i (2× magnification, illuminated with 360–400 nm light which excites GFP fluorescence). One representative image was chosen per group. **B. **Duration of survival of HSV-1 infected *scid *mice treated with PBS or CyP (n = 7 each) or control IgG or anti-asialo GM1 (n = 5 each), as compared to uninfected, CyP-treated *scid *mice (n = 7). Control IgG and anti-asialo GM1 treatment groups initially contained n = 7 mice, but two mice per group were sacrificed on day 5 p.i. for flow cytometry to determine the efficacy of NK cell depletion.

The innate resistance of *scid *mice to HSV-1 infection was not adversely affected by NK cell depletion, but was impaired by CyP-induced depletion of total WBCs (Table I). To assure that inter-experimental variance was not the source of these differences, the effect of NK cell versus total WBC depletion was directly compared in *scid *mice infected with KOS-GFP, a GFP-expressing recombinant virus [50]. *Scid *mice were treated with PBS, rabbit IgG, anti-asialo GM1, or CyP and were inoculated with 2 × 10^5 ^pfu/eye HSV-1 strain KOS-GFP. GFP expression provided a measure of the extent of KOS-GFP spread in *scid *mice (Fig. 6A). Anti-asialo GM1 antibody treatment caused a >20-fold reduction in NK cell abundance, as determined in n = 2 KOS-GFP-infected *scid *mice sacrificed on day 5 p.i Despite effective depletion of NK cells, neither treatment with control rabbit IgG nor anti-asialo GM1 had a measurable effect on KOS-GFP spread in the eyes or periocular skin of *scid *mice during the first 6 days p.i. (Fig. 6A). In contrast, CyP treatment enhanced the spread of KOS-GFP into the periocular skin of *scid *mice on day 6 p.i. relative to the other treatment groups (Fig. 6A).

**Table 1:** Duration of survival of HSV-1 infected *scid *mice.

		**Treatment**^a^			
		
**Expt.**	**Virus**	**PBS**	**rabbit IgG**	**NK-depleted**^b^	**WBC-depleted**^c^
1 ^d^	KOS	15.8 ± 0.8 (n = 9) ^f^	34.9 ± 2.8 (n = 9)	23.5 ± 0.9 (n = 9)	**ND **^g^
2	KOS-GFP	19.0 ± 0.9 (n = 5)	36.8 ± 3.8 (n = 5)	35.0 ± 0.6 (n = 5)	**ND**
3 ^e^	KOS	18.1 ± 0.8 (n = 8)	**ND **	**ND**	12.1 ± 0.7 (n = 8)
4	KOS-GFP	23.7 ± 1.8 (n = 6)	**ND**	**ND**	14.0 ± 0.6 (n = 5)
					
**Summary**		**19.2 ± 1.7 (n = 28)**	**35.9 ± 1.3 (n = 14)**	**29.3 ± 5.8 (n = 14)**	**13.1 ± 0.9 (n = 15)**

^a ^BALB/c *scid *mice were treated with PBS, control rabbit IgG, rabbit anti-asialo GM1, or cyclophosphamide (CyP) as described in Materials and Methods.

^b ^BALB/c *scid *mice were treated with rabbit anti-asialo GM1.

^c ^BALB/c *scid *mice were treated with cyclophosphamide.

^d ^The results of Experiment 1 are presented in Figure 4.

^e ^The results of Experiment 3 are presented in Figure 5.

^f ^Mean ± SEM days of survival after ocular HSV-1 inoculation of *scid *mice (n= number of mice per treatment).

^g ^Not determined in this experiment.

PBS-treated *scid *mice infected with HSV-1 strain KOS-GFP survived for 22 ± 1 days p.i. (Fig. 6B). NK cell depletion with anti-asialo GM1 did not decrease the duration of survival of HSV-1 infected *scid *mice (Fig. 6B). Rather, treatment with control IgG or anti-asialo GM1 increased the duration of survival of KOS-GFP infected *scid *mice (Fig. 6B; 38 ± 3 and 35 ± 2 days p.i, respectively). In contrast, treatment with CyP significantly reduced the duration of survival of HSV-1-infected *scid *mice (Fig. 6B; p < 0.001; 14 ± 0.2 days p.i.). The reduced duration of survival was not due to CyP's toxicity, because 7 of 7 uninfected *scid *mice survived CyP treatment (Fig. 6B). Thus, while depletion of total WBCs was correlated with decreased innate resistance to HSV-1 infection, depletion of NK cells had no such effect.

### Innate resistance to HSV-1 infection is dependent on Stat 1

Stat 1 is an IFN-activated transcription factor that is essential for the intracellular response of cells to the cytokines IFN-α/β and IFN-γ [39,40]. Lymphocyte-deficient *rag2*^-/- ^mice, which were genetically *stat1*^+/+ ^versus *stat1*^-/-^, were inoculated with 2 × 10^5 ^pfu/eye HSV-1 strain KOS-GFP. As controls, wild-type strain 129 and *stat1*^-/- ^mice were also inoculated with KOS-GFP. At 24 hours p.i., GFP expression (Fig. 7A) and infectious KOS-GFP (Fig. 7B) were detected in the eyes of all mice. Between 48 and 96 hours p.i., GFP-expression steadily decreased in the eyes of strain 129 mice and *rag2*^-/- ^mice infected with KOS-GFP (Fig. 7A). In contrast, GFP expression continued to spread in the eyes of *stat1*^-/- ^mice and *rag2*^-/-^*stat1*^-/- ^mice such that 25 to 50% of the ocular surface was GFP-positive by 72 hours p.i. (Fig. 7A). Likewise, *stat1*^-/- ^and *rag2*^-/-^*stat1*^-/- ^mice shed ~300-fold more virus on day 3 p.i. than wild-type or *rag2*^-/- ^mice (Fig. 7B). This rapid response at the site of inoculation was not lymphocyte-dependent, because wild-type mice and *rag2*^-/- ^mice shed equivalent, low titers of KOS-GFP on day 3 p.i. (Fig. 7B). Viral titers were not determined in *stat1*^-/- ^mice or *rag2*^-/-^*stat1*^-/- ^mice on day 7 p.i. because the extent of ocular pathogenesis precluded a reliable measurement.

Strain 129 (wild-type) mice uniformly survived KOS-GFP infection (Fig. 7C). In contrast, 0% of *rag2*^-/- ^mice survived and their duration of survival was 25 ± 2 days p.i. Thus, the duration of survival of KOS-GFP-infected *rag2*^-/- ^mice was similar to KOS-GFP-infected *scid *mice (i.e., 21 ± 3 days; Table I). *Rag2*^-/- ^*stat1*^-/- ^mice succumbed to HSV-1 infection much more rapidly than *rag2*^-/- ^mice, and survived for only 7.8 ± 0.4 days after inoculation with KOS-GFP (Fig. 7C). Likewise, *stat1*^-/- ^mice also succumbed to HSV-1 by 7.8 ± 0.8 days p.i., presumably because the viral infection overwhelmed these mice before an adaptive immune response could be mounted. Collectively, the results indicate that innate resistance to HSV-1 infection is intimately dependent on Stat 1-induced gene expression.

## Discussion

The current study was initiated to determine if innate resistance to HSV-1 is dependent on NK cells and their capacity to deliver IFN-γ to sites of viral infection. Despite the fact that 45% of peripheral WBCs in *scid *mice are NK cells (i.e., CD3^- ^CD49b^+^; Ref. [46,47]), NK cells made no measurable contribution to innate resistance to HSV-1. Thus, the focus of this study rapidly shifted away from the effector mechanisms that NK cells use to control HSV-1 infection, and shifted towards a series of experiments to validate that lymphocyte-deficient mice indeed possess a measurable innate resistance to HSV-1. The results are discussed, as follows.

### Role of NK cells in innate resistance to HSV-1

Despite a large differential in NK cell cytotoxicity, NOD *scid *mice possessed an innate resistance to HSV-1 infection that was equivalent to BALB/c *scid *mice. One interpretation of the results is that NK cell function is not essential for innate resistance to HSV-1. However, the fact that peripheral WBCs of NOD *scid *mice lack detectable NK cell cytotoxic activity *in vitro *does not prove that NOD *scid *mice are devoid of NK cell function *in vivo*. Indeed, one-third of peripheral WBCs in NOD *scid *mice were found to be CD3^- ^CD49b^+ ^NK cells. Thus, we were hesitant to use the behavior of HSV-1 in NOD *scid *mice as the basis for concluding that NK cells play no role in innate resistance to HSV-1. Likewise, we question the validity of comparisons of HSV-1 infection in mice that exhibit "high" or "low" natural cytotoxicity *in vitro*, because there is no evidence that these mice lack NK cell function *in vivo *[21,51].

T cells that become activated in response to viral infections express the "NK cell" markers asialo GM1, NK1.1, and CD49b (i.e., antigen recognized by DX5 monoclonal antibody; Ref. [47]). Thus, depletion of asialo GM1^+ ^T cells or NK1.1^+ ^T cells may account for the capacity of anti-asialo GM1 or anti-NK1.1 to impair the resistance of BALB/c and C57BL/6 mice to HSV-1 infection [12,26,27]. Consistent with this hypothesis, anti-asialo GM1 increased ocular viral titers in BALB/c mice on days 5 and 7 p.i., but produced no such effect in *scid *mice (Fig. 4). In BALB/c *scid *mice, NK cell depletion had no effect on (a) ocular viral titers or (b) the rate of KOS-GFP spread to the periocular skin. An important caveat of the NK cell depletion experiments was that control IgG or anti-asialo GM1 had the unanticipated effect of prolonging the survival of HSV-1 infected *scid *mice. Neither IgG preparation possessed neutralizing activity or reactivity with HSV-1 antigens by ELISA. This effect raises questions about the homeostatic mechanisms and Fc-γ-receptor dependent processes that are influenced when IgG is introduced into a *scid *mouse for the first time in its life [52-55]. However, the relevant point for this study is that a >95% reduction in NK cell abundance does not impair the capacity of a *scid *mouse to limit HSV-1 spread from the site of inoculation.

### Role of peripheral WBCs in innate resistance to HSV-1

The lack of effect of NK cell depletion on innate resistance to HSV-1 is only relevant if *scid *mice possess a measurable innate resistance to HSV-1. CyP-induced depletion of total WBCs in *scid *mice was associated with **1. **increased HSV-1 spread from the site of inoculation (evident by day 6 p.i.) and a **2. **reduced duration of survival. CyP impairs the immune response of normal mice to HSV-1 [31]. This study provides the first evidence that CyP can also be used to blunt the innate immune response to HSV-1. CyP has a narrow therapeutic window; there is only a 4-fold difference between the minimum effective dose and a fatal dose. Thus, we suspect that antibody-based depletion of the relevant WBC effector(s) would cause a more profound decrease in innate resistance to HSV-1. Based on recent evidence, professional APCs such as dendritic cells may be the relevant cellular targets whose depletion accounts for CyP's capacity to impair innate resistance to HSV-1 [56,57]. Further study is needed to test this hypothesis.

### Role of Stat 1 in innate resistance to HSV-1

The biological functions of IFN-α/β and IFN-γ are dependent on the phosphorylation of Stat 1, which results in Stat 1 dimerization, nuclear translocation, and transcriptional activation of IFN-stimulated genes [39,58]. HSV-1 infection was compared in *rag2*^-/- ^mice versus *rag2*^-/-^*stat1*^-/- ^mice to determine if innate resistance to HSV-1 is dependent on Stat 1-induced gene expression. Profound differences in viral titers and viral spread were evident in *stat1*^-/- ^versus *stat1*^+/+ ^mice by 3 days p.i. The rapidity with which HSV-1 infection spread in the absence of Stat 1 (i.e., a relevant effector) underscored the remarkable lack of effect that NK cell depletion had on innate resistance to HSV-1. The defect in Stat 1 rendered *rag2*^-/- ^*stat1*^-/- ^mice incapable of limiting HSV-1 spread, and these mice succumbed to the viral infection just 7.8 ± 1 days p.i. In contrast, the weakly virulent KOS-GFP strain [50] caused a slowly progressing infection in *rag2*^-/- ^mice that was not lethal until 25 ± 2 days p.i. Thus, Stat 1 plays an integral role in innate resistance to HSV-1 infection. Likewise, a similar phenotype of uncontrolled HSV-1 spread has been observed in IFN-α/βR^-/- ^IFN-γR^-/- ^double knockout mice [22,30]. Thus, the available evidence suggests that IFN-α/β and IFN-γ are important activators of Stat 1-induced resistance to HSV-1 *in vivo*. However, further studies are necessary to determine if chemokines and pro-inflammatory cytokines are also important contributors to Stat 1-dependent innate resistance to HSV-1.

## Conclusion

It has become evident that host IFNs are essential mediators of innate resistance to HSV-1 infection. Co-activation of IFN-α/β and IFN-γ signaling pathways produces a cooperative inhibition that renders host cells highly resistant to the replication of herpesviruses [59-62]. In the case of HSV-1, IFN-γ achieves this effect by multiplying the potency with which IFN-α/β inhibits viral replication [28]. The uncontrolled spread of HSV-1 in IFN-α /βR^-/- ^IFN-γR^-/- ^mice strongly suggests that this interaction is functionally relevant *in vivo *[22,30]. However, it remains to be determined which cells of the innate immune system, if any, are responsible for the rapid delivery and secretion of IFN-γ at sites of HSV-1 infection.

Several clinical case reports indicate that NK cells (a major potential source of IFN-γ) are essential for innate defense against HSV-1 infection in humans [1-3]. Yet, NK cells do not make a measurable contribution to the innate resistance of mice to HSV-1. How does one resolve this paradox? One possibility is that the mouse model may grossly underestimate the importance of NK cells in human resistance to HSV-1. The viral ICP47 protein binds human TAP1 with an extraordinarily high-affinity, and thus renders human cells MHC class I-bare and susceptible to NK cell-mediated lysis *in vitro *[4,8,63]. However, ICP47 binds *mouse *TAP1 with ~100-fold lower affinity than *human *TAP1, and does not efficiently disrupt MHC class I transport in mouse cells [64]. Thus, while NK cells may be integral to the mechanisms by which the human immune system recognizes HSV-infected cells (i.e., MHC class I-bare), the parallel mechanism may simply be non-functional in mice. Therefore, we conclude that while **1. **NK cells are dispensable for the innate resistance of mice to HSV-1 infection, **2. **further investigation is necessary to determine what role, if any, NK cells play in human resistance to HSV-1 infection.

## Methods

### Viruses, cells, and mice

The wild-type HSV-1 strains KOS [65] and KOS-GFP, a KOS strain engineered to express GFP [50], were generously provided by Dr. Priscilla Schaffer and Dr. John Balliet (Harvard University Medical School, Boston, MA). The viruses were propagated in Vero cells (American Type Culture Collection, Manassas, VA) and stored as viral stocks at -70°C until used. Vero cells were propagated in Dulbecco's modified Eagle medium (DMEM) containing 10% fetal bovine serum (FBS), hereafter referred to as "complete DMEM." YAC-1 cells (American Type Culture Collection) were propagated in RPMI-1640 containing 10% FBS.

Female BALB/c, BALB/c *scid*, and NOD *scid *mice were obtained from the Jackson Laboratory (Bar Harbor, ME). Female strain 129 mice, *rag 2*^-/- ^mice, *stat1*^-/- ^mice, and *rag2*^-/- ^*stat1*^-/- ^mice were purchased from Taconic Farms (Germantown, NY). These studies were reviewed and approved by an IACUC, and animals were handled in accordance with the *NIH Guide for the Care and Use of Laboratory Animals*. Prior to ocular inoculation, mice were anaesthetized by intraperitoneal (i.p.) administration of xylazine (6.6 mg/kg) and ketamine (100 mg/kg). Mice were inoculated by scarifying the cornea with the blunted tip of a 25-gauge needle, blotting tear film from the eyes, and by placing 4 μl of complete DMEM containing 2 × 10^5 ^pfu/eye of virus on each eye. Viral titers were determined by swabbing the ocular surface of both eyes at times after inoculation with a cotton-tipped applicator. The tip of the applicator was removed, incubated in 0.4 ml complete DMEM for 1 hour on ice, and viral titers were determined in the supernatant by a microtiter plate plaque assay. Following anaesthetization of mice, fluorescent images of mouse eyes infected with HSV-1 strain KOS-GFP were taken at 2× or 4× magnification on a Nikon TE300 fluorescent microscope (Nikon Instruments, Lewisville, TX).

### Flow cytometric analysis of natural killer cells and T lymphocytes

Randomly chosen BALB/c, BALB/c *scid*, and NOD *scid *mice were tested to confirm that they were deficient for T- and B-cell function. Serum from mice was tested for immunoglobulin G (IgG) levels using an ELISA kit specific for the Fc fragment of mouse IgG (Bethyl Laboratories, Montgomery, TX). Flow cytometric analysis was used to measure the abundance of CD4^+ ^T cells and CD8^+ ^T cells in the spleens of selected mice, as described below.

Cells were harvested from the spleens of mice and red blood cells were removed by hypotonic lysis in 0.84% NH_4_Cl. WBCs were labeled with fluorescent-labeled monoclonal antibodies obtained from BD Biosciences (San Jose, CA) according to the manufacturer's directions. For each mouse analyzed, 1 × 10^6 ^WBCs were labeled with either **1. **nothing, **2. **fluorescein-isothiocyanate (FITC)-labeled anti-CD3 (clone 17A2) + phycoerythrin (PE)-labeled anti-CD4 (clone GK1.5), **3. **FITC-labeled anti-CD3 + PE-labeled anti-CD8 (clone Ly-2), or **4. **FITC-labeled anti-CD3 + PE-labeled anti-CD49b (clone DX5). Additionally, controls were included for gating and compensation, and these included WBCs labeled with **5. **nothing, **6. **FITC-labeled IgG_2b _isotype control (clone G27-35), **7. **PE-labeled IgG_2b _isotype control (clone G27-35), **8. **PE-labeled IgG_2a _isotype control (clone G155-178), or **9. **PE-labeled IgM isotype control (G155-258). Flow cytometry was performed immediately after antibody labeling on a FACSCalibur using CellQuest Pro software (BD Biosciences). A minimum of 10,000 events was recorded per sample. When NK cell depletion was monitored in mice, a minimum of 25,000 events were recorded per sample. The threshold between fluorescence-positive and -negative was set such that >99.5% of WBCs incubated with control antibodies were considered negative.

### Adoptive transfer of lymphocytes to BALB/c scid mice

Unfractionated and purified WBCs were obtained from naïve BALB/c donors for adoptive transfer to BALB/c *scid *mice, as follows. Spleens and cervical lymph nodes were removed from ten naïve BALB/c mice and dissociated to yield a single cell suspension. WBCs were purified using Lympholyte M according to the manufacturer's directions (CedarLane Laboratories Ltd., Hornby, Ontario, Canada). Purified lymphocytes were obtained by passing BALB/c spleen WBCs through immunoaffinity B cell and T cell columns according to the manufacturer's directions (Cytovax Biotechnologies Inc., Edmonton, Alberta, Canada). Adoptive transfer of WBCs was achieved by intravenous (i.v.) tailvein injection of BALB/c *scid *mice with 0.5 ml complete RPMI-1640 containing nothing (vehicle), 5 × 10^6 ^unfractionated WBCs (total WBCs), 5 × 10^6 ^purified B cells, or 5 × 10^6 ^purified T cells.

### NK cell cytotoxicity assay

YAC-1 cells (1 × 10^4 ^cells) were labeled with ^51^Cr and incubated with BALB/c, BALB/c *scid*, and NOD *scid *spleen WBCs in round bottom 96-well plates for 6 hours at 37°C at effector : target ratios of 100:1, 50:1, 25:1, 12.5:1, and 6.25:1 (n = 3 per group). One hundred microliters of supernatant were collected from each culture for determination of ^51^Cr release from target cells. Controls for the assay included 1 × 10^4 ^cells target cells incubated alone in culture medium (spontaneous release) and target cells incubated with 0.5% Triton X-100 (maximal release). The percent cytotoxicity in each group of three replicate cultures was calculated, as follows:



### NK cell depletion and cyclophosphamide treatment

Between days -1 and 10 p.i., BALB/c mice and BALB/c *scid *mice were given four to six i.p. injections of normal rabbit IgG (Rockland Immunochemicals, Gilbertsville, PA) or rabbit IgG containing anti-asialo GM1 antibody (Wako Chemicals USA, Richmond, VA). The efficacy of NK cell depletion was validated by flow cytometric comparison of NK cell (CD3^- ^CD49b^+^) frequency. Cyclophosphamide (Mead Johnson Oncology Products, Princeton, NJ) was diluted with phosphate-buffered saline (PBS) such that a volume of 0.25 ml delivered i.p. would achieve a dose of 125 mg/kg (e.g., 11 mg/ml for 22 g mice). Vehicle-treated controls received 0.25 ml PBS. Intraperitoneal injections of PBS or cyclophosphamide were administered on days -1, +1, and +3 after viral inoculation.

### Statistical analysis

Analysis of numerical data and statistical analyses were performed with the software packages Microsoft Excel (Redmond, WA) and Modstat (Modern Microcomputers, Mechanicsville, VA). Unless otherwise indicated, all data are presented as means ± standard errors of the means (SEM). Viral titers were transformed by adding a value of 1 to the number of pfu per eye such that negative results (i.e. no plaques detected) could also be analyzed on a logarithmic scale. The significance of differences in viral titers between three or more groups was statistically evaluated by one way analysis of variance (ANOVA) followed by Tukey's post hoc t-test. The significance of differences in duration of survival between each treatment group and PBS-treated controls was evaluated by a two-way t-test.

## Competing interests

The author(s) declare that they have no competing interests.

## Authors' contributions

BMG carried out the *scid *vs NOD *scid *experiments and NK cell cytotoxicity assays. WPH carried out the anti-asialo GM1 and CyP depletion experiments with the assistance of JLM. JLM performed the flow cytometric analysis of spleen cells. WPH and BMG conceived of the study. WPH wrote the manuscript.
